# Supplementary material for: Identification of enzymatic functions of osmo-regulated periplasmic glucan biosynthesis proteins from Escherichia coli reveals a novel glycoside hydrolase family
Source: Commun Biol. 2023 Sep 21;6:961. doi: 10.1038/s42003-023-05336-6 (PMC10514313; doi:10.1038/s42003-023-05336-6)
Supplement: Supplementary file 2 — Supplemental information [file 42003_2023_5336_MOESM2_ESM.pdf]

Supplemental information for

Identification of enzymatic functions of osmo-regulated periplasmic glucan biosynthesis proteins from *Escherichia coli* reveal a novel glycoside hydrolase family

Sei Motouchi, Kaito Kobayashi, Hiroyuki Nakai & Masahiro Nakajima*

*Masahiro Nakajima

E-mail: m-nakajima@rs.tus.ac.jp

This file includes:

Supplemental Table 1 to 2

Supplemental Figures 1 to 13

Supplementary Note

Supplementary Table 1 | Residues interacting with substrates of EcOpgD and EcOpgG

**Subsite**	**Residues**	
	EcOpgD	EcOpgG
**−9**	(No substrate observed)	(Subsite −7)
**−8**	(No substrate observed)	K144 (g)
**−7**	R171, R172	K134, E147
**−6**	(Subsites −4 and −7)	D146; (main) E147, Y166
**−5**	D173; (main) Y190	Y56
**−4**	R162[a], D173[b], **R184**[c], **Y192**	Y56 (g)[d], Y83, K134[a], E147[b], **R158**, **Y166**
**−3**	Q191, **R359**	D327, **R332**
**−2**	N82, W441	
**−1**	**R184**, **D300**, **R359**, **D388***(g)	**R158**, **D273**, **R332**, **D361***(g)
	(main) **E385**, L387	(main) **E358**, N360
**+1**	**R184**, **S195**; (main) **L178**	**R158**, **S169**; (main) **L152**
**+2**	E445; (main) **T386**	(main) **T359**
**+3**	**F211**, **Y182**	**F185**, **Y156**
**+4**	**Y182**, **R197**, **E209**, **D388***	**Y156**, **R171**, **E183**, **D361***
	(main) **D388***	(main) **D361***
**+5**	(Subsites +3 and +6)	(Subsites +3 and +7)
**+6**	(Subsites +4 and +5)	E183
**+7**	(No substrate observed)	(Subsites +2, +5, and +6)

[a, b], Chemically conserved residues between EcOpgD and EcOpgG.

[c], Conserved residues between EcOpgD and EcOpgG are shown in bold letters.

[d], (g) represents hydrogen bonds with glycosidic bond oxygen atoms. Anomeric hydroxy groups are used to describe the positions of the subsites.

Asterisks (*) represent catalytic residues substituted with asparagine residues to obtain the substrate complexes.

Double underlines represent hydrophobic interactions with the substrates.

Subsite positions in parentheses represent intramolecular hydrogen bonds with Glc moieties at the subsites.

Supplementary Table 2 | Primers used in this study.

	Forward	Reverse
EcOpgD		
Cloning (wild-type)	TTTAAGAAGGAGATATACATATGGCAGATTCTGATATTGCCGACGG	GTGGTGGTGGTGGTGGTGCTCGAGACTCATCACGCGGTCGTCAACAT
P68A	ATGACGGCGCAGGCTTATAACAGTATT	AGCCTGCGCCGTCATTGTCGCCAGCGT
P68L	ATGACGCTGCAGGCTTATAACAGTATT	AGCCTGCAGCGTCATTGTCGCCAGCGT
Y346F	GGCTGGTTTAACAAACGCCCAAGTCTG	TTTGTTAAACCAGCCCATAATGTCCTG
R349A	AACAAAGCCCCAAGTCTGTGGGTGGAA	ACTTGGGGCTTTGTTATACCAGCCCAT
T376L	GGCGAACTGCTGGATAACATTGTCTGC	ATCCAGCAGTTCGCCCGTTGTTGGGAT
T376A	GGCGAAGCGCTGGATAACATTGTCTGC	ATCCAGCGCTTCGCCCGTTGTTGGGAT
W431F	GAAGGTTTCGCGCCAGGTGAACACTAT	TGGCGCGAAACCTTCCGAGAAACCGCC
W431L	GAAGGTTTGGCGCCAGGTGAACACTAT	TGGCGCCAAACCTTCCGAGAAACCGC
E199Q	AGTAAACAAGAGTTCCCCGACTTTACC	GAACTCTTGTTTACTGTCGGTGTAAGT
D290N	ATTCATAACTCTGATCGTCTGTCCATG	ATCAGAGTTATGAATTTGCGGATGAAT
D341N	TATCAGAACATTATGGGCTGGTATAAC	CATAATGTTCTGATAATGGGAGAAGTC
E375Q	ACGGGCCAAACGCTGGATAACATTGTC	CAGCGTTTGGCCCGTTGTTGGGATTTC
D378N	ACGCTGAATAACATTGTCTGCTTCTGG	AATGTTATTCAGCGTTTCGCCCGTTGT
E429Q	TTCTCGCAAGGTTGGGCGCCAGGTGAA	CCAACCTTGCGAGAAACCGCCCATGCC
E435Q	CCAGGTCAACACTATCCCGAAAAATGG	ATAGTGTTGACCTGGCGCCCAACCTTC
EcOpgG		
Cloning (wild-type)	TTTAAGAAGGAGATATACATATGTTCAGTATTGATGATGTCGCAAAG	GTGGTGGTGGTGGTGGTGCTCGAGTTCATTGGCAGGTAACTGGTAGC
D361N	ACCAACAATAACATCGTCGCTTACTGG	GATGTTATTGTTGGTTTCATCGTTGGT

All primer pairs are represented from 5′ to 3′.

Forward primers for wild-type were designed to eliminate N-terminal signal sequences predicted by signalP5.0 server[47].

The positions of mutations are underlined.

Supplementary Figure 1 | pH and temperature profiles.

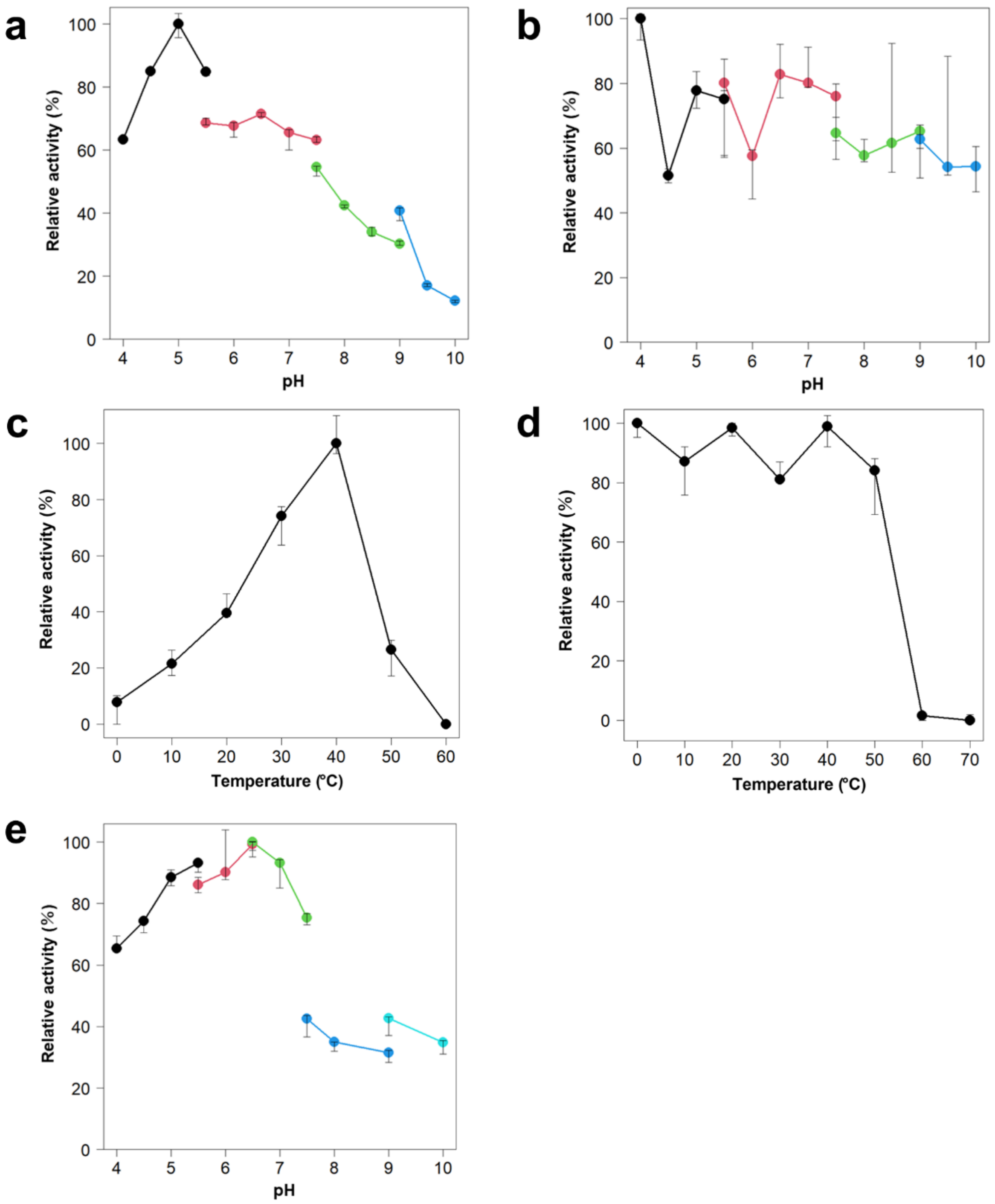

a, Optimum pH of EcOpgD. Buffers used for the enzymatic reactions were sodium acetate-HCl (pH 4.0–5.5, black), Bis-Tris-HCl (pH 5.5–7.5, red), Tris-HCl (pH 7.5–9.0, green) and glycine-NaOH (pH 9.0–10, blue). **b**, pH stability of EcOpgD. Buffers used for incubating the enzyme were the same as (**a**). **c**, **d**, temperature optimum (**c**) and stability (**d**). **e**, Optimal pH of EcOpgG. Buffers used for the enzymatic reaction were sodium acetate-HCl (pH 4.0–5.5, black), MES (pH 5.5–6.5, red), MOPS (pH 6.5–7.5, green), Tris-HCl (pH 7.5–9.0, blue) and glycine-NaOH (pH 9.0–10, cyan). Medians of triplicate experiments were plotted. Other data were used to show error bars.

Supplementary Figure 2 | Elution profiles of standards, EcOpgD and EcOpgG on SEC.

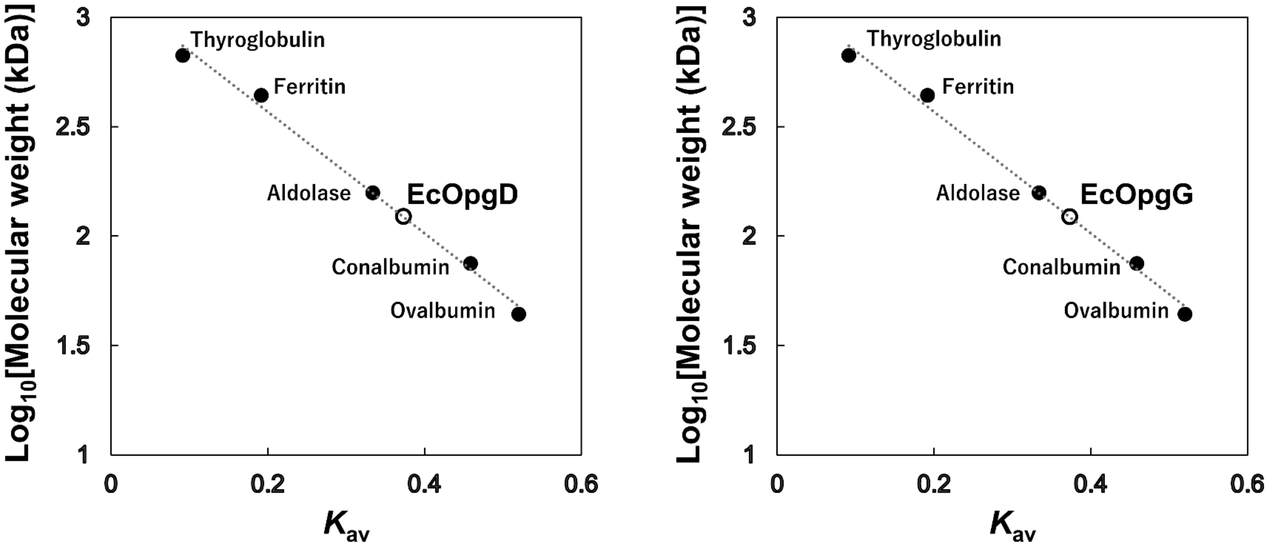

Closed and circles represent the standard protein markers, and ligand-free EcOpgD and EcOpgG, respectively. The K_{av} values of the enzymes are 0.37 and 0.37, respectively. Regression lines are shown as dotted lines.

Supplementary Figure 3 | Superposition between ligand-free EcOpgD and EcOpgG.

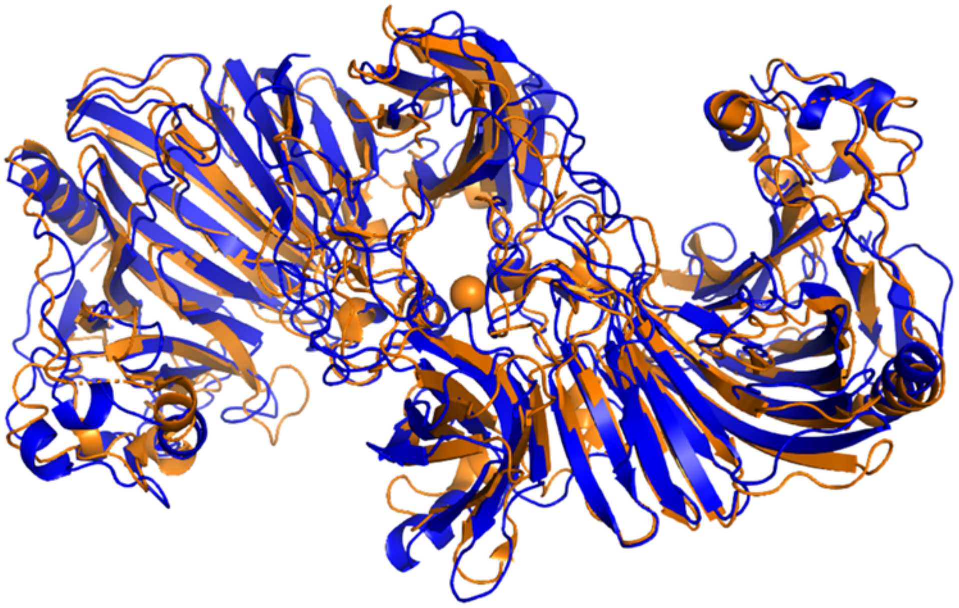

The ligand-free structure of EcOpgD (Chains A and B) and EcOpgG (PDB ID; 1txk) are shown as blue and orange cartoons, respectively. A sodium ion in the ligand-free structure of EcOpgG is shown as an orange sphere.

Supplementary Figure 4 | Multiple alignment of EcOpgD and EcOpgG.

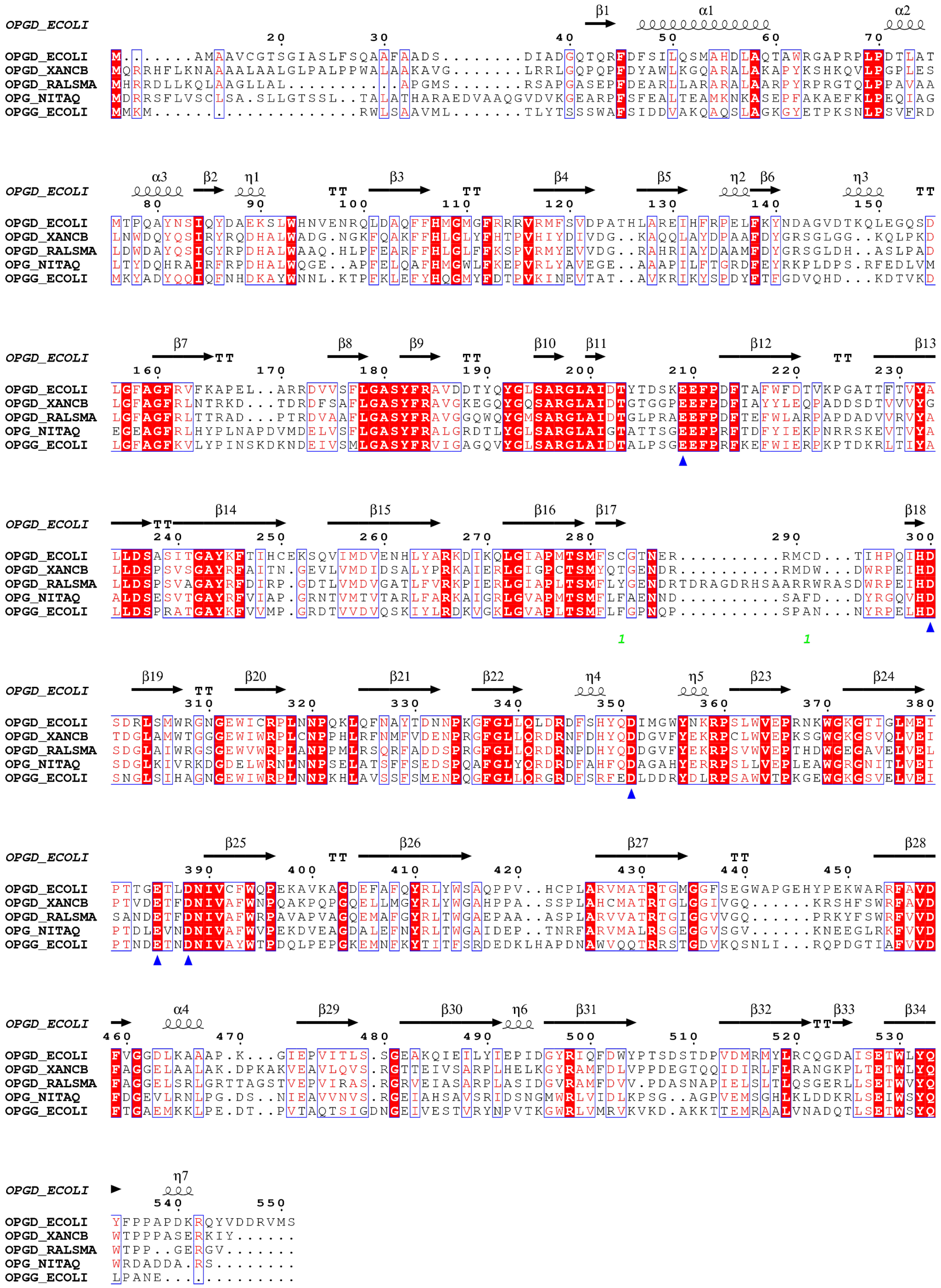

The structure-based multiple alignment was performed by PROMALS3D[62] and visualized using the ESPript 3.0 server (http://espript.ibcp.fr/ESPript/ESPript/)[63]. Secondary structures of EcOpgD are shown above the sequences. Blue triangles are shown below all acidic residues conserved in this alignment and are located at the cleft in the ligand-free structures of EcOpgD or EcOpgG.

Supplementary Figure 5 | Electron densities of substrates in the Michaelis complex of EcOpgD and EcOpgG.

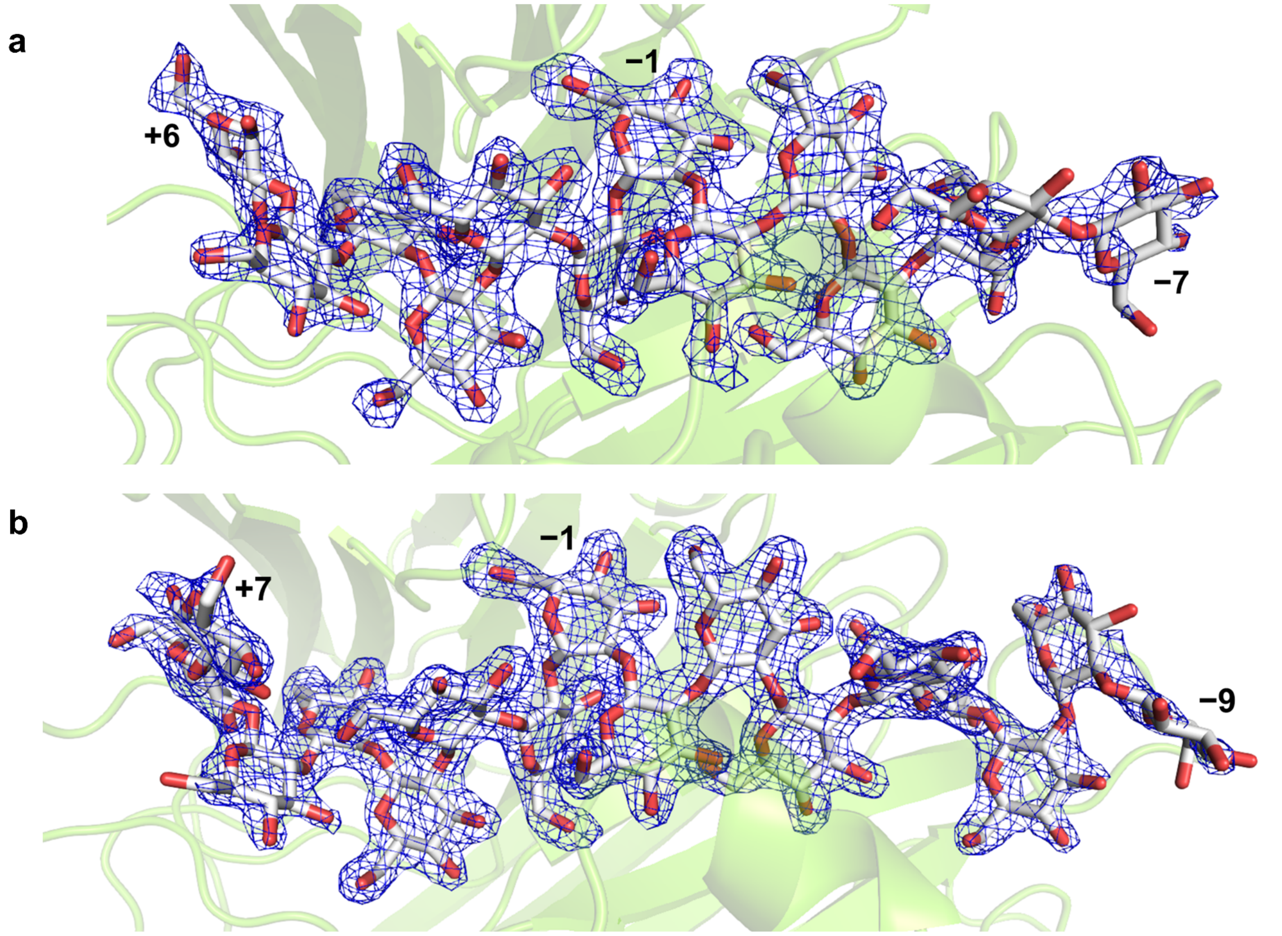

a, EcOpgD. **b**, EcOpgG. Substrates are shown as white sticks. Each chain B is shown as a semi-translucent light green. The electron densities of β-1,2-glucans are shown as F_o–F_c omit maps by blue meshes at the 3σ contour level.

Supplementary Figure 6 | Substrate binding site of EcOpgD forming a cleft in the ligand-free structure and a tunnel in the Michaelis complex structure.

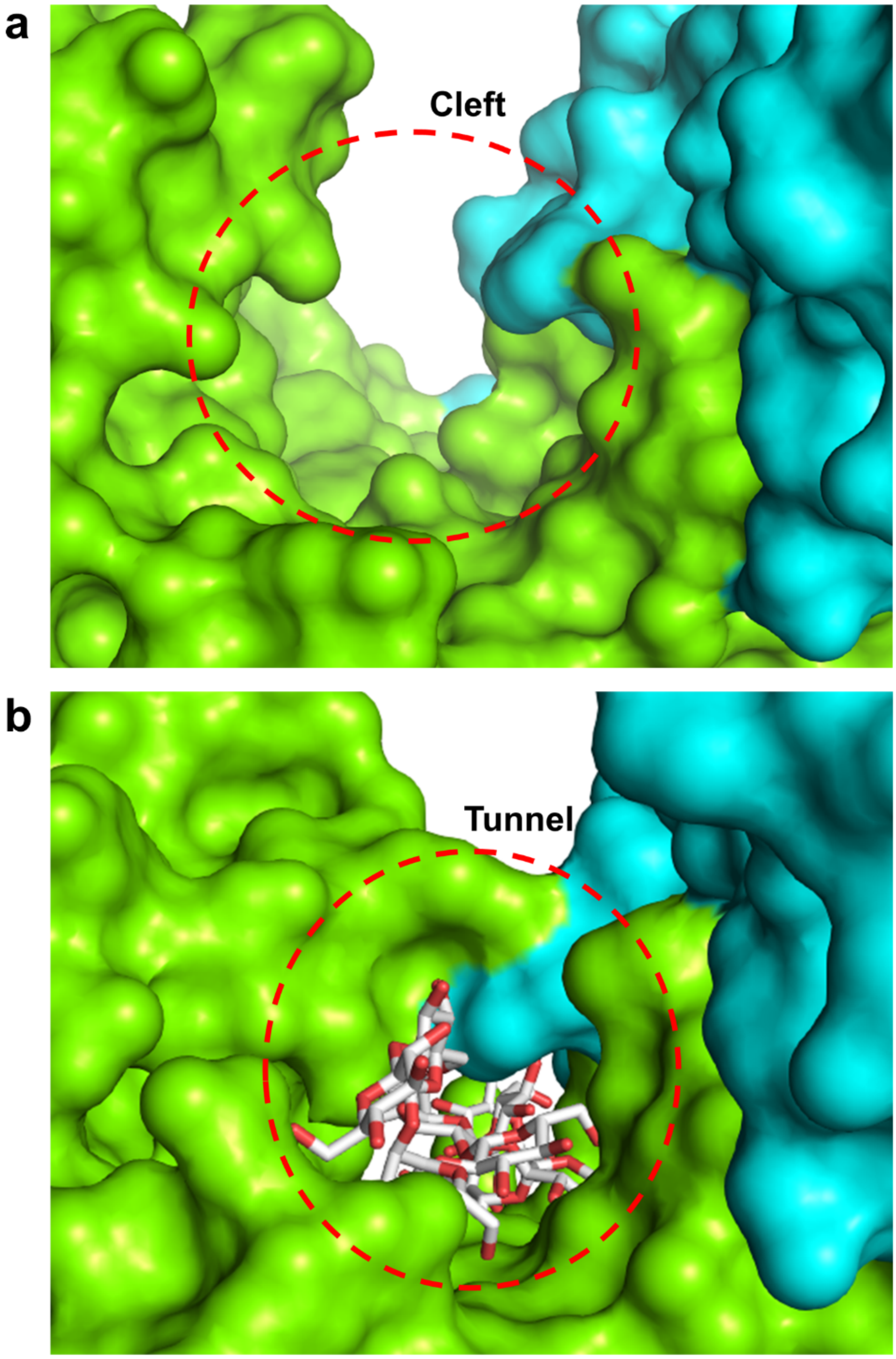

a, A cleft in the ligand-free structure. **b**, A tunnel in the Michaelis complex. Chains A and B are shown as cyan and light green surfaces, respectively. The substrate is shown as a white stick. Red dotted circles in (**a**) and (**b**) represent a cleft and a tunnel of the substrate binding sites, respectively.

Supplementary Figure 7 | Electron densities around the Glc moieties at subsites −1 (a) and −5 (b) of EcOpgD.

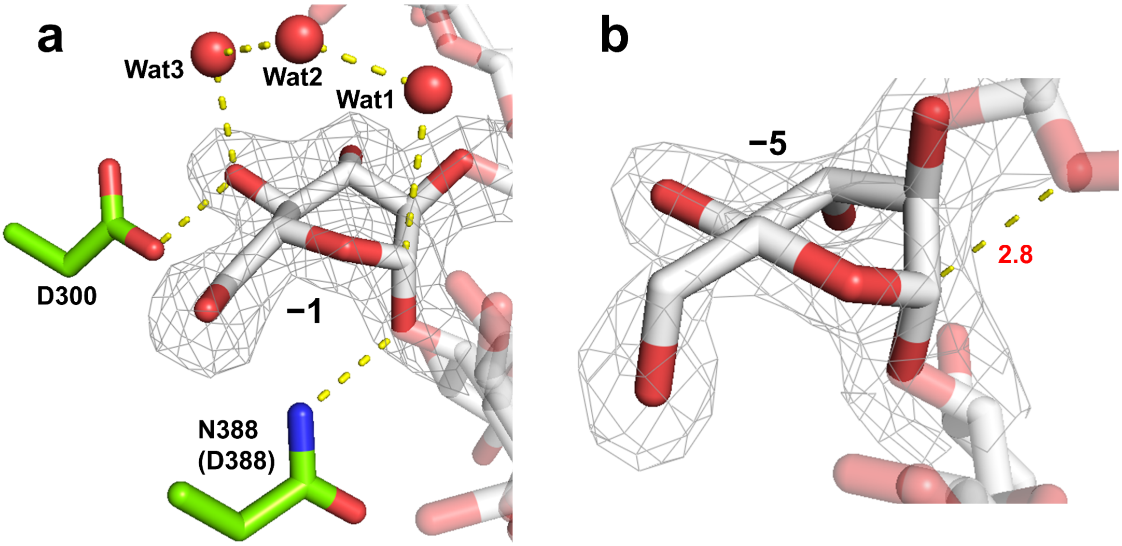

Substrates and amino acid residues in Chain B are shown as white and light green sticks, respectively. Wat1, Wat2 and Wat3 are shown as red spheres. Hydrogen bonds are shown as yellow dashed lines. The electron densities of the Glc moieties at subsites −1 and −5 are shown as F_o–F_c omit maps by gray meshes at the 3σ contour level.

Supplementary Figure 8 | Substrate recognition residues in the Michaelis complexes.

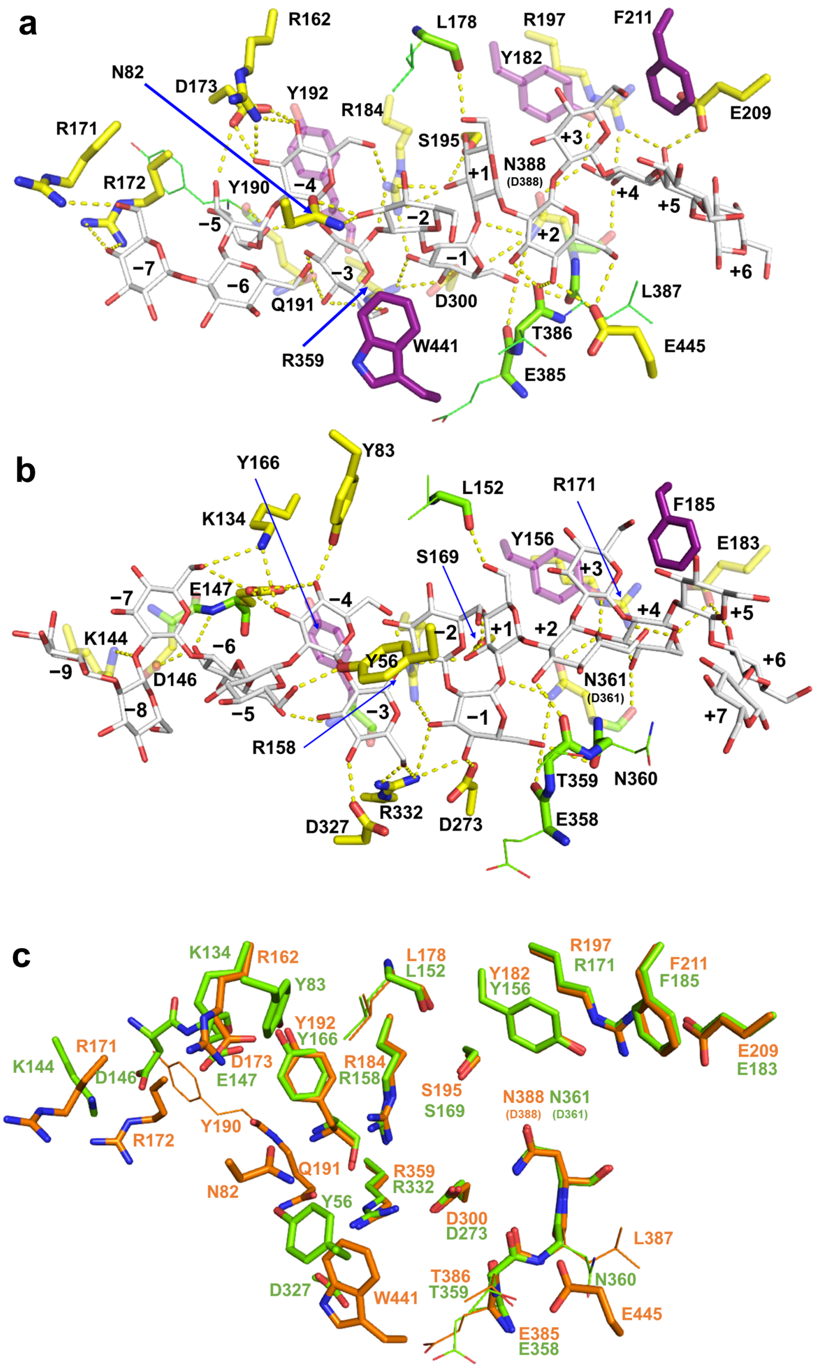

a, **b**, Complexes of EcOpgD D388N (**a**) and EcOpgG D361N (**b**). Substrates are shown as white sticks. The main chains and side chains of residues interacting with substrates by their main chains are shown in light green sticks and lines, respectively. Residues forming hydrogen bonds with the substrates through their side chains are shown as yellow sticks. Residues forming hydrophobic interactions with the substrates are shown as purple sticks. Residues behind the substrates at this view are shown semi-translucently. Blue arrows are used to label particular amino acids. **c**, Superposition of substrate recognition residues between EcOpgD and EcOpgG. Residues of EcOpgD and EcOpgG are shown in orange and light green, respectively. The use of lines and stick models is based on (**a**, **b**).

Supplementary Figure 9 | CD spectra of wild-type and mutants of EcOpgD.

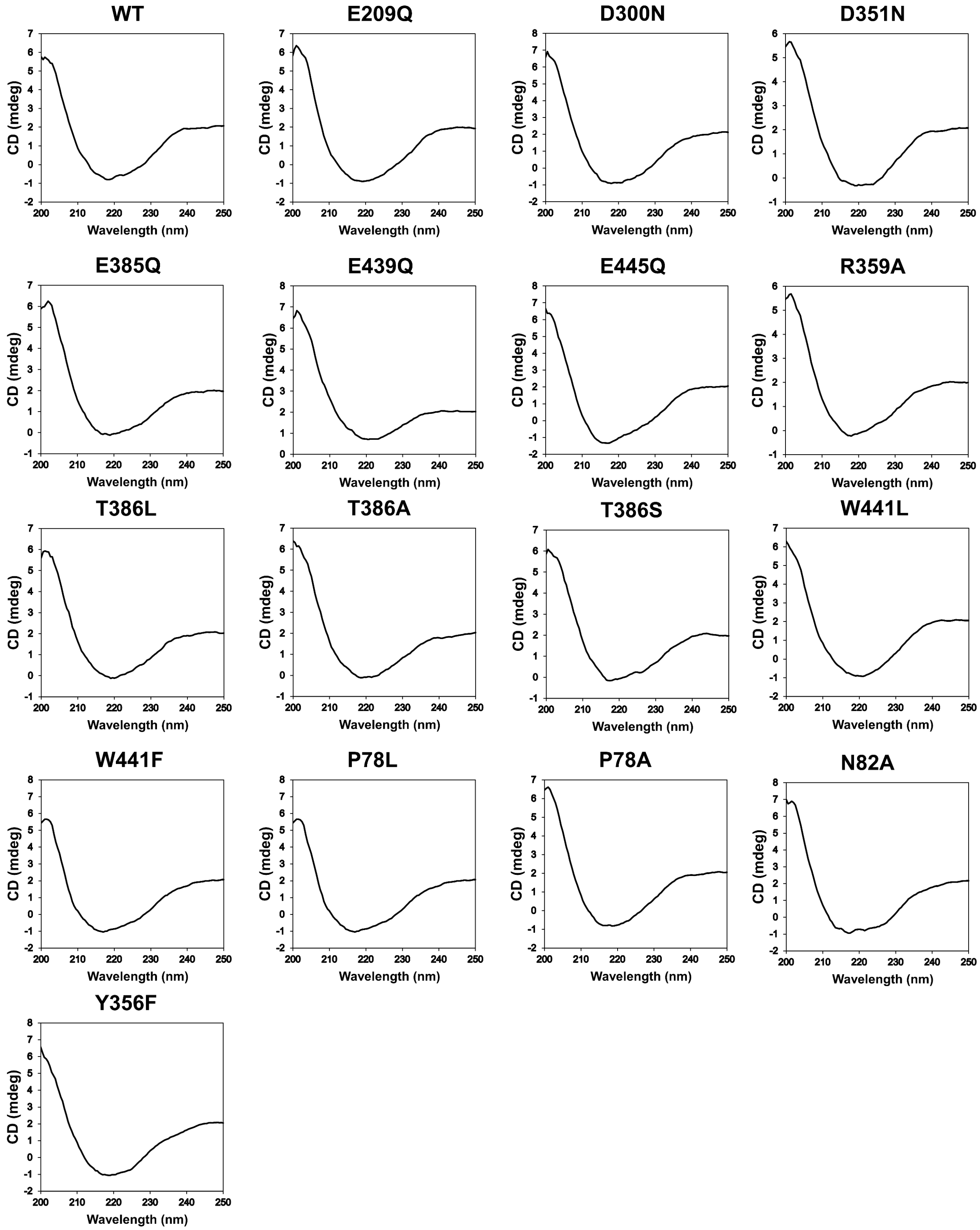

Supplementary Figure 10 | Interaction of Loop A with surroundings in the EcOpgD complex structure.

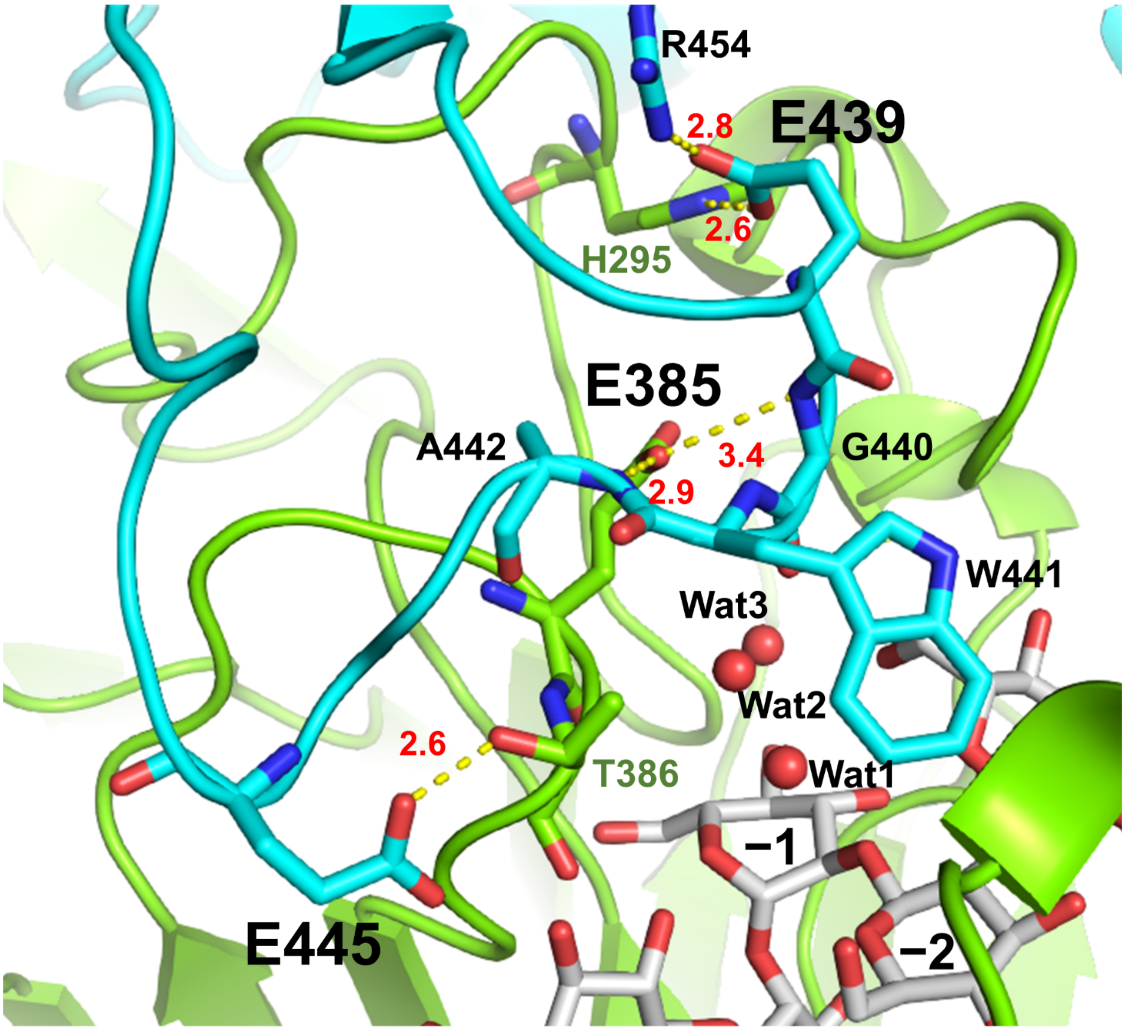

Chains A and B are shown in cyan and light green, respectively. Hydrogen bonds with distances (Å, red numbers) are shown as yellow dotted lines. Substrates are shown as white sticks. All residues used for the fixation of Loop A are shown as sticks. E385 interacting with the main chain nitrogen atoms of G440 and A442, E439 interacting with H295 and R454, and E445 interacting with T386 are labeled with large bold letters. Residues in chain B are labeled in green letters. Wat1, Wat2 and Wat3 are shown as red spheres.

Supplementary Figure 11 | Spaces for β-1,6-side chains at minus and plus subsites of EcOpgD and EcOpgG.

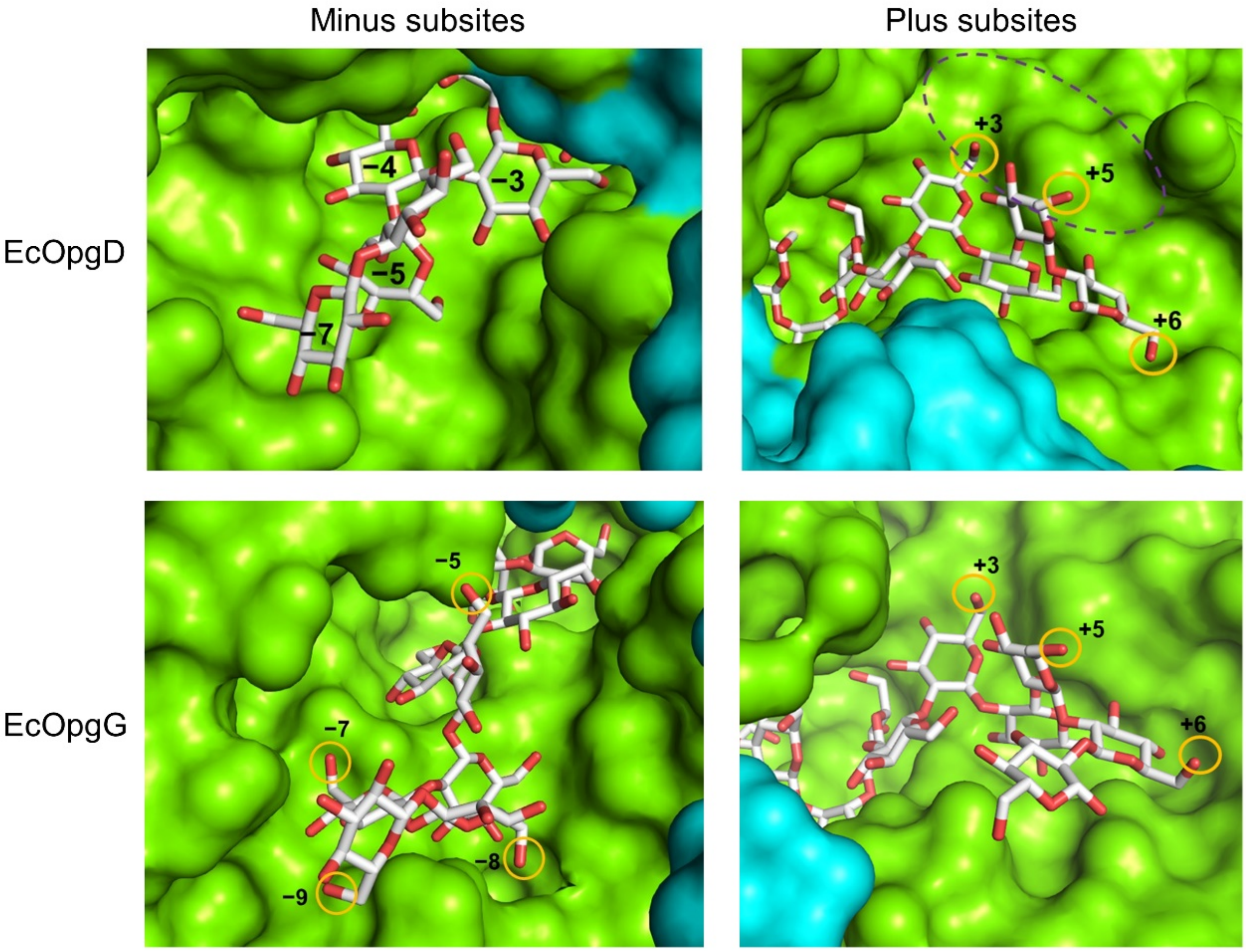

Chains A and B are shown in cyan and light green surfaces, respectively. Substrates are shown as white sticks. The 6-hydroxy groups of Glc moieties at the subsites, which clearly have sufficient space for Glc side chains, are indicated by orange circles. The large space of EcOpgD that accommodates β-1,6-linked side chains is indicated as a dotted circle.

Supplementary Figure 12 | Phylogenetic tree of the MdoG superfamily.

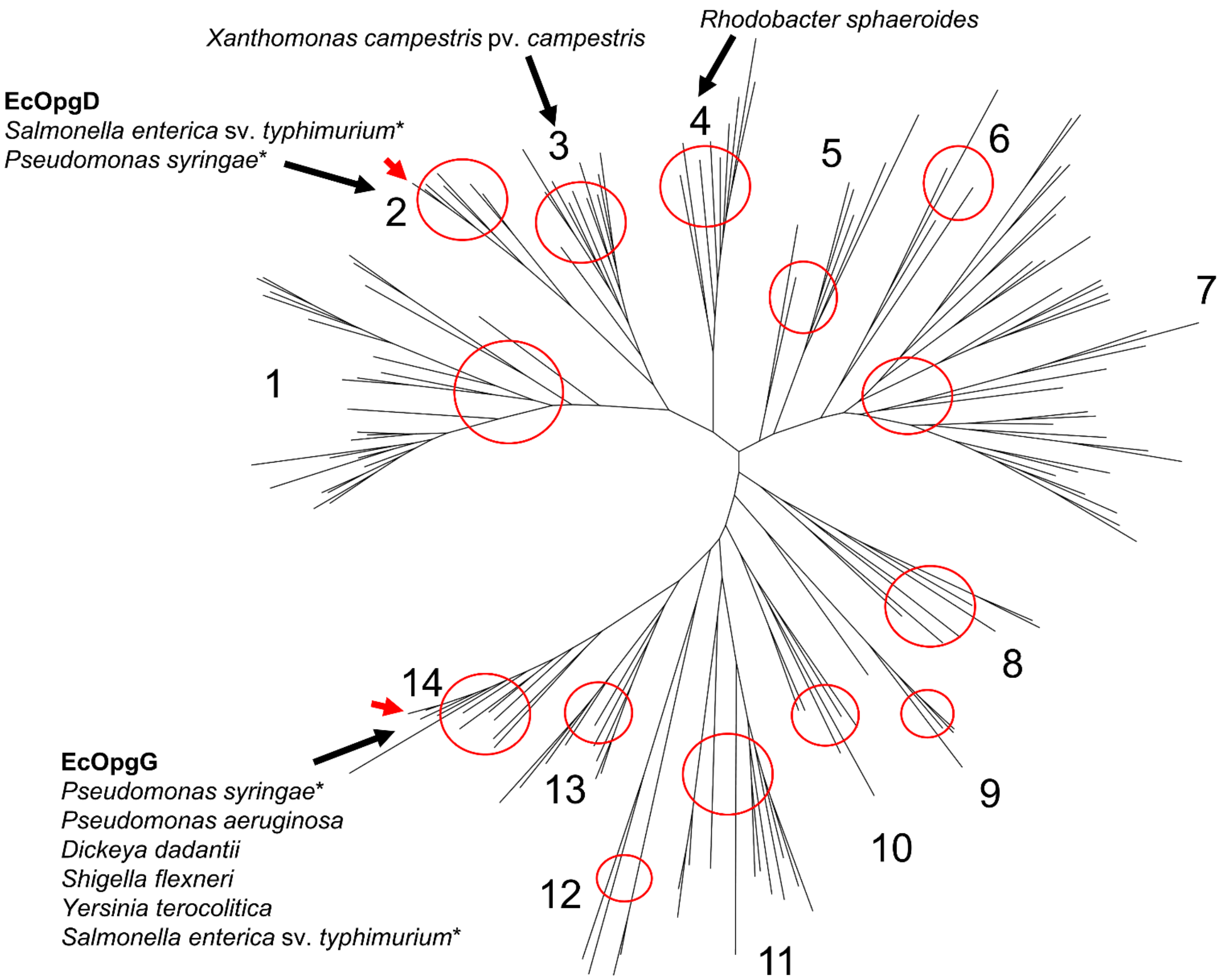

Each clade is indicated by a red circle with a clade number. Black arrows indicate clades, including homologs from Gram-negative bacteria whose phenotypes of OPG-related genes knockout mutants have been investigated. Asterisks represent species possessing two homologs. Red arrows indicate the branches of EcOpgD and EcOpgG.

Supplementary Figure 13 | Conservation of residues among the MdoG superfamily.

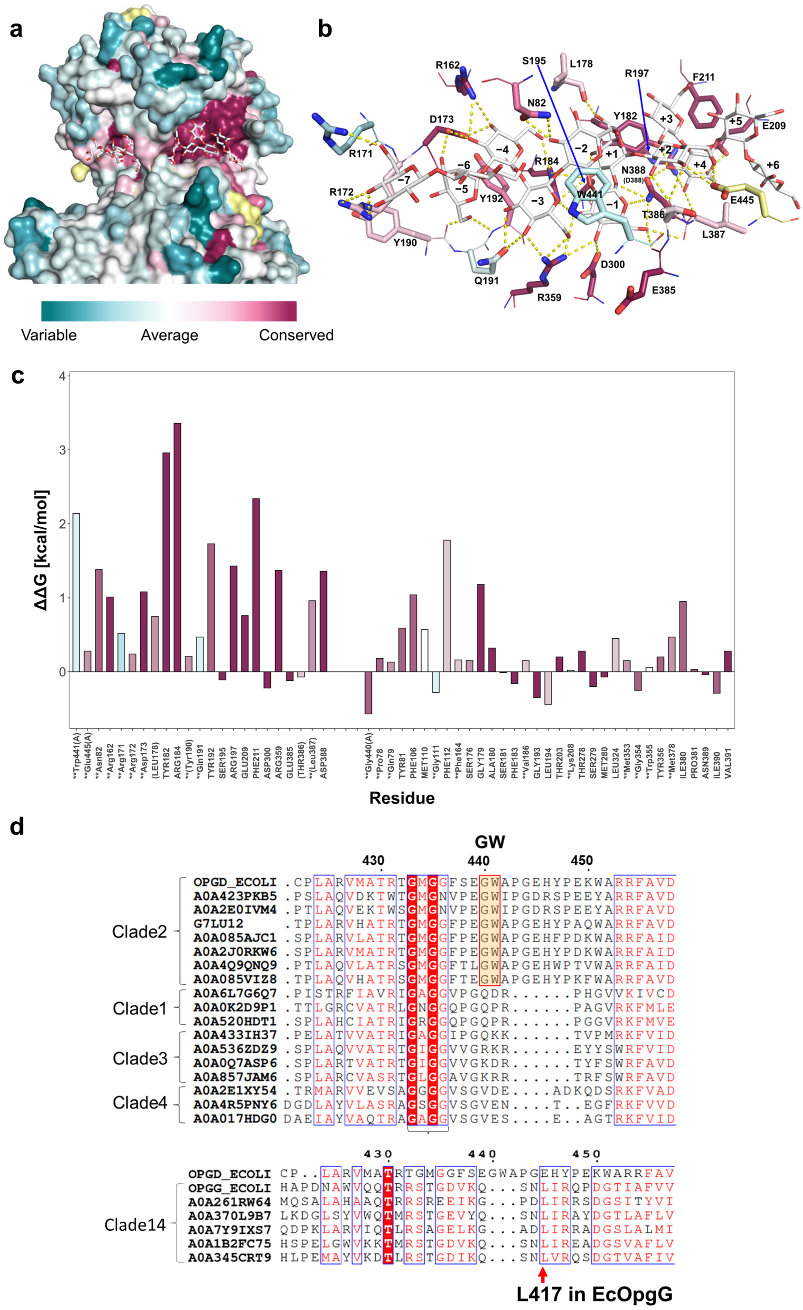

a, **b**, Conserved residues of EcOpgD. Surface model (**a**) and substrate recognition residues (**b**) of the EcOpgD-β-1,2-glucan complex colored based on the color bar corresponding to conservation scores. The conservation scores were calculated by ConSurf using EcOpgD. **c**, Contribution of residues for substrate binding. Residues shown on the left and right sides are substrate recognition residues and residues in close proximity to the substrate rather than substrate recognition, respectively. The colors used for the bars are the same as (**a**, **b**). Parentheses represent the main chains that recognize the substrate. Residues in chain A are indicated with (A). Asterisks denote residues that are not conserved in EcOpgG. **d**, Multiple alignments of clades 1–4 and 14. Multiple alignments of homologs in clades 1–4 and 14 was visualized using the ESPript 3.0 server (http://espript. ibcp.fr/ESPript/ESPript/)[64]. The homologs are represented by UniProt accession numbers. Residue numbers of EcOpgD are shown above the alignment.

Supplementary Note

Biological assembly of EcOpgG

Hanoulle et al. describes that EcOpgG is a monomer in solution[26]. However, the article does not show any result or method for determining the assembly of EcOpgG. In addition, there is no evidence that EcOpgG is a monomer as far as the authors know. The binding dissociation energy of EcOpgG dimer calculated by PISA suggested that the stable assembly of EcOpgG is a dimer both in the presence and absence of a sodium ion at the interface of the two subunits (13.5 kcal/mol, 3.0 kcal/mol, respectively). However, the sodium ion highly contributes to the dimer assembly according to the dissociation energy. To confirm the true assembly, we performed the size-exclusion chromatography (SEC) for ligand-free EcOpgG in the presence of 100 mM NaCl and found that EcOpgG completely assembled as a dimer. Although we also tried to perform SEC analysis in the absence of NaCl, most of EcOpgG precipitated when the concentration of NaCl decreased. Therefore, we avoided performing SEC analysis in the absence of NaCl. Considering that *opgGH* genes are expressed in response to hypo-osmotic condition in *E. coli*[1], a sodium ion might be one of the keys for assembly of EcOpgG.
